# Real-World Anthropometric Outcomes, Safety, and Use Patterns of GLP-1 and Dual GIP/GLP-1 Receptor Agonists in Obesity and Glucose Metabolism Disorder: A Nationwide Multicentre Community Pharmacy Study

**DOI:** 10.3390/healthcare14193355

**Published:** 2026-10-08

**Authors:** Olatz Vergniory-Trueba, Carlos Treceño-Lobato

**Affiliations:** 1 Pharmaceutical Care España Foundation, 08009 Barcelona, Spain; 2 ADViSe Research Group, Universidad Europea Miguel de Cervantes (UEMC),47012 Valladolid, Spain

**Keywords:** GLP-1 receptor agonists, obesity, type 2 diabetes mellitus, community pharmacy, real-world evidence, pharmacovigilance, semaglutide, tirzepatide

## Abstract

**Highlights:**

**What are the main findings?**

- Incretin-based therapies were associated with substantial observed weight reductions in routine clinical practice, although these estimates were based partly on retrospectively recalled baseline weight.
- Nearly three-quarters of patients achieved at least 5% weight loss and more than half achieved at least 10%, indicating clinically meaningful observed weight-loss outcomes in this real-world sample.
- Gastrointestinal adverse events were common but crude safety signals lost statistical significance after multiple-testing correction, with older age being the only independent predictor of fewer digestive side effects association after adjustment for measured covariates.

**What are the implications of the main findings?**

- These findings provide real-world evidence of substantial observed weight reductions among patients receiving incretin-based therapies in community pharmacy practice.
- The results support the role of community pharmacies in monitoring patients receiving incretin-based therapies, including treatment adherence, observed weight outcomes, and reported suspected adverse drug reactions, and provide a rationale for the development and future evaluation of a standardized pharmacy-led dispensing and follow-up protocol.

**Abstract:**

Background: Glucagon-like peptide-1 receptor agonists (GLP-1RAs) have transformed the management of obesity and type 2 diabetes mellitus (T2DM). Although randomized clinical trials have demonstrated substantial weight loss and metabolic benefits with incretin-based therapies, real-world evidence is needed to characterize anthropometric outcomes, safety, prescribing patterns, and treatment use under routine clinical conditions. Objectives: The objective of this study was to characterize real-world anthropometric outcomes, safety, and use patterns of GLP-1 receptor agonists and dual GIP/GLP-1 receptor agonists in patients with obesity or glucose metabolism disorder receiving treatment through Spanish community pharmacies. Methods: A nationwide multicentre cross-sectional study with retrospective data collection was conducted between January and April 2026 across community pharmacies in Spain. Adult patients receiving GLP-1 receptor agonists or dual GIP/GLP-1 receptor agonists were consecutively enrolled and classified according to therapeutic indication (obesity or glucose metabolism disorder). Changes in body weight and body mass index (BMI) were assessed using paired-sample *t*-tests, while multivariable linear regression explored independent determinants of weight loss. Crude safety signals were initially evaluated using exploratory crude safety association analysis based on Odds Ratios (ORs). Subsequently, multivariable logistic regression was performed to identify independent predictors of suspected digestive adverse drug reactions after adjustment for demographic characteristics, treatment-related variables, lifestyle factors, and concomitant glucose-lowering therapies. Baseline body weight was retrospectively reported by participants and was not independently verified against clinical records or other objective sources, whereas current body weight was measured by the pharmacist at the study visit. Results: A total of 531 patients were included (52.2% with obesity; 47.8% were classified in the glucose metabolism disorder (GMD) cohort, comprising 200 patients with type 2 diabetes mellitus and 54 with prediabetes/fasting glucose disturbance; the mean age was 56.5 ± 12.5 years; and 66.7% were women). The mean observed reduction in body weight from treatment initiation to the study assessment was 12.17 ± 10.64 kg, corresponding to an 11.71% ± 9.40% reduction relative to retrospectively reported baseline weight. Overall, 73.9% and 54.1% of participants achieved ≥5% and ≥10% weight loss, respectively. No statistically significant unadjusted difference in relative weight loss was detected across treatment groups; however, equivalence was not formally assessed. Gastrointestinal suspected adverse drug reactions were the most frequently reported safety outcomes. Exploratory crude analyses showed nominal associations between Wegovy^®^ and nausea/vomiting and between Mounjaro^®^ and constipation, as well as between concomitant insulin and dizziness and SGLT2 inhibitor therapy and urinary disorders; however, none of these associations remained statistically significant after Benjamini–Hochberg correction for multiple testing. In the exploratory multivariable linear regression model, treatment duration, treatment agent, dietary adherence, light physical activity, and age showed statistically significant adjusted associations with observed percentage weight loss. In the multivariable logistic regression model, increasing age was the only variable significantly associated with lower odds of reported suspected digestive adverse drug reactions (adjusted OR = 0.97 per year; 95% CI 0.96–0.99; *p* = 0.003). Conclusions: Observed percentage weight loss varied across treatment groups, although between-treatment comparisons should be interpreted cautiously because of differences in treatment duration, therapeutic indication, sample size, and dose exposure. Given the retrospective ascertainment of baseline weight and the cross-sectional design, these findings should be interpreted as real-world estimates of observed weight change rather than causal estimates of treatment effectiveness. Reported suspected adverse drug reactions were common, particularly gastrointestinal events. Although several nominal associations were observed in exploratory crude analyses, none remained statistically significant after correction for multiple testing. These findings support the importance of individualized monitoring in community pharmacy settings. These findings provide a rationale for the prospective development and evaluation of a standardized pharmacy-led dispensing and follow-up protocol to support patient safety and treatment adherence.

## 1. Introduction

Glucagon-like peptide-1 receptor agonists (GLP-1RAs) and dual glucose-dependent insulinotropic polypeptide (GIP)/GLP-1 receptor agonists, such as tirzepatide, represent a major advance in the pharmacological management of obesity and T2DM. By mimicking endogenous incretin hormones, these agents enhance glucose-dependent insulin secretion, suppress glucagon release, delay gastric emptying, and increase satiety, leading to clinically meaningful weight loss and metabolic effects [1,2].

Large randomized clinical trials, including the STEP, SURPASS, and SURMOUNT programmes, have demonstrated the efficacy of semaglutide and tirzepatide in achieving substantial weight reduction [3,4]. However, participants in clinical trials are highly selected and managed under controlled conditions, which may limit the generalizability of these findings to routine clinical practice [5]. Consequently, real-world evidence (RWE) is needed to evaluate the effectiveness and safety of these therapies in everyday healthcare settings. In addition, limited evidence is available regarding prescribing patterns, dose-escalation practices, treatment interruptions related to drug shortages, and adherence to lifestyle recommendations in routine care [6].

Community pharmacies are well positioned to generate RWE because of their accessibility, frequent patient contact, and role in medication monitoring. In Spain, this research capacity is supported by collaborative initiatives such as the Sentinel Pharmacy Networks (Redes de Farmacias Centinela), which have contributed to pharmacovigilance and post-marketing drug safety surveillance across a range of therapeutic areas [7,8,9,10].

Existing real-world studies in Spain have primarily evaluated individual GLP-1 receptor agonists in clinical settings. For example, multicentre Spanish studies have examined oral or subcutaneous semaglutide predominantly in patients with type 2 diabetes, using hospital- or healthcare-centre-based cohorts and focusing mainly on metabolic and anthropometric outcomes. More recent real-world evidence from Spanish primary care has also evaluated GLP-1 receptor agonists in relation to metabolic outcomes and health-related quality of life [11,12].

In other European settings, population-based studies have begun to characterize treatment patterns and patient experiences with GLP-1 receptor agonists, but these studies have generally focused on specific agents or selected aspects of treatment use [13].

The present study differs from this existing evidence in several respects. First, it was conducted entirely through a nationwide network of community pharmacies, providing a pharmacy-based perspective on incretin therapy during routine dispensing and pharmaceutical care. Second, it included multiple GLP-1 receptor agonists and the dual GIP/GLP-1 receptor agonist tirzepatide across patients treated for obesity and type 2 diabetes. Third, the study integrated observed anthropometric outcomes with the systematic recording of reported suspected adverse drug reactions, treatment duration, dose-escalation and use patterns, concomitant glucose-lowering therapies, and lifestyle-related variables within the same assessment. Finally, the study extends a previously piloted community pharmacy methodology from two Spanish pharmacies to a broader multicentre network, although the participating pharmacies were convenience-sampled and should not be considered nationally representative [14].

Community pharmacy-based assessment may provide complementary information to clinical records because pharmacists have repeated contact with patients at the point of medication dispensing and can integrate anthropometric measurements with information obtained during medication review and patient counselling. Previous community pharmacy-based weight-management studies have demonstrated the feasibility of obtaining objectively measured body weight and BMI during routine pharmacy encounters and using these measures to evaluate changes over time [15]. A recent systematic review also identified a substantial body of evidence evaluating pharmacist-led weight-management interventions using changes in body weight, BMI, or waist circumference as clinical outcomes, with most studies conducted in community pharmacy settings [16]. This setting may therefore provide an opportunity to capture treatment-related outcomes together with medication use, adherence-related information, reported adverse reactions, and lifestyle factors that may not be systematically available in routine clinical records.

Although randomized clinical trials provide robust evidence under controlled conditions, evidence from routine community pharmacy practice remains comparatively limited, particularly for studies integrating observed anthropometric outcomes with the characterization of reported suspected adverse drug reactions, treatment-use patterns, and concomitant therapies. Previous real-world studies have provided important information on the effectiveness, persistence, and safety of incretin-based therapies, but much of this evidence has been generated from clinical or administrative datasets rather than from structured pharmacist-led assessments in community pharmacy settings [3,4,5,6]. In Spain, community pharmacy networks offer an accessible setting for systematic medication monitoring and generation of real-world evidence, but nationwide evidence using this setting to characterize incretin-based therapies remains scarce [7,8,9,10]. Building on our previous pilot study conducted in Spanish community pharmacies [14], the present study was designed to address this gap by providing nationwide multicentre data on observed anthropometric outcomes, reported suspected adverse drug reactions, treatment-use patterns, and factors associated with observed weight-loss outcomes.

The primary objective of this study was to quantify the observed percentage change in body weight between the retrospectively reported weight at treatment initiation and the body weight measured at the study assessment among adults receiving incretin-based therapies in routine community pharmacy practice. Secondary objectives were to describe absolute body-weight change and BMI change; characterize the distribution of treatment use, including treatment duration, dose-escalation patterns, and concomitant glucose-lowering therapies; describe reported suspected adverse drug reactions; compare observed anthropometric outcomes across treatment and clinical indication subgroups; and explore factors associated with observed percentage weight loss.

To address these objectives, a nationwide multicentre study was conducted through a network of Spanish community pharmacies coordinated by the Pharmaceutical Care España Foundation.

## 2. Materials and Methods

### 2.1. Study Design and Setting

A nationwide multicentre cross-sectional study with retrospective data collection was conducted in Spanish community pharmacies. The primary outcome was the observed percentage change in body weight from treatment initiation to study assessment, calculated from the retrospectively reported treatment-initiation weight and the body weight measured by the community pharmacist at the study visit. Secondary outcomes included absolute body-weight change, change in BMI, achievement of ≥5% and ≥10% weight loss, treatment-use patterns, reported suspected adverse drug reactions, and factors associated with observed percentage weight loss. Because baseline body weight was retrospectively reported and not independently verified, these outcomes were interpreted as observed real-world changes rather than prospectively measured treatment effects.

The study methodology and electronic data collection tools were based on those previously developed and validated in a pilot study [14].

Participating community pharmacies were recruited using convenience sampling through professional networks and institutional dissemination channels. The study questionnaire was disseminated through all Official Colleges of Pharmacists in Andalusia, as well as through the Official Colleges of Pharmacists of Bizkaia, Álava, Palencia, and Tenerife. Additional participation was facilitated through individual collaborating members of the Pharmaceutical Care España Foundation. Because recruitment was conducted through institutional and professional dissemination channels rather than through a centralized individual invitation to pharmacies, the exact number of pharmacies approached and the total number of pharmacies that received the invitation cannot be reliably established, and a pharmacy-level recruitment rate could therefore not be calculated. Within participating pharmacies, eligible patients were consecutively invited to participate during routine dispensing and pharmaceutical care activities.

Data collection was performed during a point-in-time pharmaceutical interview conducted over a 4-month window from January 2026 to April 2026.

#### Sample Size and Recruitment Target

No formal a priori statistical sample-size calculation was performed. Instead, the recruitment target was defined operationally at the pharmacy level, with each participating community pharmacy requested to recruit three patients with obesity and three patients with glucose metabolism disorder. This recruitment strategy was established to obtain a balanced representation of the two clinical indication cohorts across participating pharmacies. The final sample size was determined by the number of eligible patients recruited during the predefined study period. A total of 537 patients were initially recruited, of whom 6 did not provide informed consent, resulting in a final study population of 531 participants.

Because the recruitment target was not based on a prespecified effect size, prevalence estimate, statistical power, or anticipated loss to follow-up, no formal assumptions for these parameters were specified.

### 2.2. Study Population and Eligibility Criteria

The study population consisted of adult patients who visited any of the participating community pharmacies and were receiving incretin-based therapy during the study window. Patients were enrolled consecutively during routine medication dispensing and pharmaceutical care activities. No minimum treatment duration was required for study inclusion. Patients were eligible regardless of the duration of incretin-based therapy at the time of assessment, including patients at treatment initiation and those receiving treatment for more than 12 months. Treatment duration was recorded as a study variable and was subsequently considered in the analysis of observed weight-loss outcomes. Of the 537 patients initially recruited, 6 did not provide informed consent and were therefore excluded from the study. The remaining 531 participants constituted the final study population. Six participants did not complete the questionnaire and were excluded from questionnaire-based analyses.

Participants were classified into two mutually exclusive cohorts according to their documented clinical conditions and anthropometric characteristics. Patients with obesity, defined according to the study eligibility criterion by a body mass index (BMI) > 27 kg/m^2^, were classified into the obesity cohort. This classification also applied to patients with diabetes mellitus when their BMI was >27 kg/m^2^. Patients with documented type 2 diabetes mellitus or prediabetes/fasting glucose disturbance who did not meet this obesity criterion were classified into the glucose metabolism disorder (GMD) cohort. Thus, in patients with coexisting obesity and a glucose metabolism disorder, the obesity cohort classification took precedence when BMI was >27 kg/m^2^. Cohort assignment was based on clinical and anthropometric criteria and was not determined by the treatment product received. Comorbidities and relevant clinical conditions were recorded during the standardized pharmacist-led interview based on the information documented in the study questionnaire.

**Obesity Cohort:** Patients receiving therapies approved for weight management, specifically Wegovy^®^ (semaglutide) or Mounjaro^®^ (tirzepatide), were included.

**Glucose Metabolism Disorder Cohort:** Patients receiving incretin-based therapies for the management of T2DM or other glucose metabolism disorders, including Ozempic^®^ (semaglutide), Rybelsus^®^ (oral semaglutide), Trulicity^®^ (dulaglutide), and Victoza^®^ (liraglutide), were included.

Exclusion criteria comprised (i) age under 18 years; (ii) current pregnancy or lactation; and (iii) refusal to participate.

### 2.3. Variables and Data Collection

Data were systematically recorded by trained community pharmacists during the point-of-care interview using a standardized electronic case report form (eCRF).

Variables were grouped into the following categories:

Sociodemographic Data: Patient age, biological sex, and geographic location (province) were recorded.

Clinical and Anthropometric variables: Documented comorbidities, current body weight (kg) measured at the pharmacy using calibrated digital scales, height (m), and waist and hip circumferences (cm) obtained by trained pharmacists utilizing standardized non-stretchable anthropometric tapes were recorded.

Baseline body weight was retrospectively reported by participants at the study visit and was not independently verified against clinical records, previous pharmacy measurements, or other objective sources. Current body weight was measured by the community pharmacist at the time of assessment. Accordingly, the anthropometric outcomes were calculated from a retrospectively reported treatment-initiation weight and an objectively measured weight at study assessment.

Observed percentage weight loss was calculated as [(retrospectively reported treatment-initiation weight − weight at study assessment)/retrospectively reported treatment-initiation weight] × 100.

Treatment and Prescribing Patterns: Active molecule, current dose, administration frequency (weekly, daily, or others), total treatment duration, history of previous exposure to other incretins, specific dose-escalation practices, and patient-reported administration difficulties were recorded. Prescriber specialty and prescription type (public or private) were also recorded.

Concomitant Pharmacotherapy: To evaluate concomitant medications and metabolic interactions within routine clinical practice, active concomitant medications were systematically recorded. These were categorized into specific pharmacological classes, explicitly auditing the use of Metformin, sodium-glucose cotransporter-2 inhibitors (SGLT2i/gliflozins), exogenous insulin formulations, and sulfonylureas.

Lifestyle Interventions: Concomitant patient adherence to structured dietary modifications and regular physical activity patterns (categorized as sedentary, light, moderate, or intense) since treatment initiation were recorded.

Dietary and physical activity variables were assessed through patient self-report using the standardized study questionnaire. No validated dietary adherence or physical activity instrument was used. Dietary adherence was recorded according to whether the patient reported following dietary recommendations related to the treatment, and was coded as no/yes (0/1) for the main analysis. In addition, patients were asked whether they had changed their diet or physical activity since treatment initiation.

Physical activity was assessed through patient self-report using the standardized study questionnaire. Participants were considered to meet the physical activity criterion when they reported performing at least 150 min of physical activity per week. Physical activity intensity was categorized as light, moderate, or intense, with examples provided in the questionnaire to facilitate classification. Light activity included activities such as walking, yoga, and household chores; moderate activity included brisk walking, swimming, and cycling; and intense activity included competitive sports, vigorous aerobic exercise, and heavy physical work. These categories were based on the operational definitions provided in the study questionnaire and were not derived from a validated physical activity assessment instrument or objective activity monitoring.

Physical activity was therefore considered a self-reported lifestyle variable, with the 150-min-per-week threshold used as the study criterion for meeting the physical activity recommendation.

Safety Outcomes: Adverse drug reactions (ADRs) were assessed retrospectively from treatment initiation to the study assessment through an active pharmacist-led interview using the standardized study questionnaire. During the pharmacy interview, patients were systematically asked about symptoms potentially associated with their incretin-based treatment, including gastrointestinal, neurological, urinary, musculoskeletal, and other predefined symptom categories. Reported events were recorded by the community pharmacist, and when applicable, information regarding severity, management, referral, and follow-up was documented. No formal validated causality assessment tool, such as the Naranjo algorithm or the WHO-UMC criteria, was systematically applied. Accordingly, the recorded events represent patient-reported suspected adverse drug reactions occurring during treatment and were not considered causally confirmed drug-induced events.

These were systematically categorized according to MedDRA system organ classes, including digestive (specifically isolating nausea/vomiting and constipation), nervous (dizziness), cardiovascular, dermatological, musculoskeletal, metabolic, and genitourinary systems, as well as general systemic disorders (asthenia).

Qualitative Observations: Free-text comments regarding treatment experience, drug shortages, or tolerability were also recorded.

### 2.4. Statistical Analysis

The primary outcome was observed percentage change in body weight, calculated as [(weight at treatment initiation − weight at study assessment)/weight at treatment initiation] × 100. Primary outcome analyses included descriptive estimation of mean percentage weight loss and its standard deviation, together with the proportion of participants achieving clinically relevant thresholds of ≥5% and ≥10% weight loss. Exploratory analyses examined differences in observed percentage weight loss across treatment-duration categories, treatment groups, clinical indication subgroups, and concomitant medication groups. Multivariable linear regression was used to explore factors associated with observed percentage weight loss.

Continuous variables were expressed as mean ± standard deviation (SD) or median [interquartile range] depending on their distribution, verified using the Shapiro–Wilk normality test. Categorical variables were described using absolute frequencies and percentages with their corresponding 95% confidence intervals (95% CI).

Paired-sample *t*-tests were used to compare retrospectively reported treatment-initiation body weight and body weight measured at the study assessment, and to compare the corresponding BMI values. These tests were used to assess whether the observed within-participant differences differed statistically from zero. Because treatment-initiation weight was retrospectively reported and the study used a cross-sectional design, the resulting *p*-values were not interpreted as evidence of a prospectively observed or causal treatment effect.

The study recorded the current treatment dose at the time of assessment, rather than the maximum dose achieved or the complete dose-escalation trajectory.

The primary anthropometric outcome was the observed percentage change in body weight, calculated from the retrospectively reported treatment-initiation weight and the weight measured at study assessment.

Responsiveness to treatment was evaluated categorically by calculating the proportion of patients achieving established clinically relevant weight-loss thresholds (≥5% and ≥10% cumulative weight loss). Anthropometric weight-loss outcomes and categorical response rates were stratified according to pharmacological active agent, commercial brand, and baseline therapeutic indication (obesity versus glucose metabolism disorder). To compare observed percentage weight loss across treatment agents, one-way ANOVA followed by Tukey’s Honestly Significant Difference (HSD) post-hoc test for multiple comparisons was performed.

Differences in observed percentage weight loss across treatment groups were assessed using one-way analysis of variance (ANOVA). Homogeneity of variances was assessed using Levene’s test. When the assumption of homogeneity of variances was satisfied, Tukey’s honestly significant difference (HSD) test was used for post-hoc pairwise comparisons. If heterogeneity of variances was detected, Welch’s ANOVA with Games–Howell post-hoc comparisons was used instead. These procedures were selected to account for the unequal sample sizes across treatment groups.

To explore factors independently associated with observed percentage weight loss while adjusting for measured covariates included in the model, an exploratory multivariable linear regression model was constructed using cumulative percentage weight loss as the primary continuous dependent variable. Independent variables included baseline sociodemographics, active brand selection, structured lifestyle compliance, exposure chronology, and concomitant glucose-lowering drugs.

An exploratory multivariable linear regression model was fitted to examine factors associated with observed percentage weight loss. The model included therapeutic indication (obesity vs. glucose metabolism disorder), age, sex, treatment group, dietary adherence, physical activity level, treatment duration, and concomitant use of SGLT2 inhibitors, metformin, insulin, and sulfonylureas. Therapeutic indication was included as the main variable of interest to assess whether the observed difference in percentage weight reduction between the obesity and glucose metabolism disorder cohorts persisted after adjustment for demographic, treatment-related, lifestyle, and concomitant medication variables.

Model diagnostics included assessment of functional form using the Ramsey RESET test, residual normality using the Shapiro–Wilk test, homoscedasticity using the Breusch–Pagan test, influential observations using Cook’s distance, leverage, and externally studentized residuals, and multicollinearity using variance inflation factors (VIFs). Because heteroscedasticity was detected, HC3 heteroscedasticity-robust standard errors were used for inference. No observations were excluded solely on the basis of regression diagnostic statistics.

Comparisons of observed weight-loss outcomes according to concomitant glucose-lowering therapy (metformin, sodium–glucose cotransporter 2 [SGLT2] inhibitors, insulin, and sulfonylureas) were considered exploratory. For each concomitant medication comparison, an independent-sample *t*-test was used to compare observed percentage weight loss between exposed and non-exposed participants. Four separate comparisons were performed. *p*-values were reported as nominal and unadjusted, and no multiplicity correction was applied. Given the potential for confounding by clinical indication and patient characteristics, these comparisons were not interpreted as independent or causal treatment effects.

Treatments represented by fewer than five participants (Saxenda^®^ and Victoza^®^) were included in the descriptive characterization of the study population but excluded from drug-specific comparative analyses and multivariable regression models because of insufficient sample size for reliable estimation.

Given the substantial variability in treatment duration, observed weight-loss outcomes were additionally examined according to treatment-duration categories: treatment initiation, ≤3 months, 3–6 months, 6–12 months, and >12 months. These analyses were considered exploratory and were intended to characterize the observed anthropometric outcomes across different stages of treatment exposure rather than to estimate a causal time-dependent treatment effect.

To identify independent factors associated with suspected digestive adverse drug reactions, a multivariable logistic regression analysis was performed. The multivariable logistic regression model showed a likelihood ratio χ^2^ of 25.4 (*p* = 0.034) and a McFadden’s pseudo-R^2^ of 0.044, indicating limited overall explanatory performance. Digestive adverse drug reactions (yes/no) were considered the dependent variable, while age, sex, GLP-1RA agent, treatment duration, physical activity, dietary intervention, and concomitant glucose-lowering therapies (metformin, sodium-glucose cotransporter-2 inhibitors [SGLT2i], insulin, and sulfonylureas) were included as prespecified independent variables based on their clinical relevance. Adjusted odds ratios (aORs) with corresponding 95% confidence intervals (95% CIs) were estimated. Statistical significance was defined as a two-sided *p*-value < 0.05.

Given the large number of exploratory crude safety comparisons, *p*-values from these analyses were adjusted for multiple testing using the Benjamini–Hochberg procedure to control the false discovery rate (FDR). The FDR correction was applied jointly to all 133 exploratory crude safety comparisons. Associations with an FDR-adjusted *p*-value < 0.05 were considered statistically significant. Crude ORs were retained for descriptive and exploratory purposes and interpreted as observed real-world associations rather than evidence of independent or causal drug-specific effects.

Overall model fit was assessed using the likelihood ratio test (LR χ^2^) and McFadden’s pseudo-R^2^, which were interpreted as measures of overall model fit and explanatory performance. Formal assessment of model discrimination and calibration was not performed.

Data curation was performed using Microsoft Excel for Microsoft 365 (Microsoft Corporation, Redmond, WA, USA), and statistical analyses were performed using Stata version 19 (StataCorp LLC, College Station, TX, USA). All statistical tests were two-tailed.

Missing data were handled using an available-case approach. No missing values were imputed, and multiple imputation was not performed. For descriptive and univariable analyses, all participants with available data for the variable under analysis were retained; consequently, the analytical denominator varied slightly across variables according to data completeness. For multivariable regression analyses, a complete-case (listwise) approach was used, whereby participants were included only when complete data were available for all variables included in the corresponding model. Therefore, the sample size of each multivariable model depended on the completeness of the variables included in that model. For analyses requiring paired measurements, only participants with complete data for both measurements were included.

### 2.5. Ethical Considerations

The study protocol was approved by the Basque Country Ethics Committee for Clinical Research (CEIm de Euskadi) under approval code EOM2025059, serving as an authorized methodological extension of the research line initiated in the pilot project [14]. The study was conducted in accordance with the ethical principles of the Declaration of Helsinki and applicable ethical and data protection regulations. All participants provided written informed consent before inclusion in the study.

In accordance with Spanish and European data protection regulations (Spanish Organic Law 3/2018 on the Protection of Personal Data and Guarantee of Digital Rights [LOPDGDD] and EU Regulation 2016/679 [General Data Protection Regulation, GDPR]), all collected data were anonymized and managed within an automated, secure database, guaranteeing absolute patient confidentiality and anonymity.

## 3. Results

### 3.1. Baseline Demographic, Clinical, and Behavioral Characteristics

**Figure 1:**
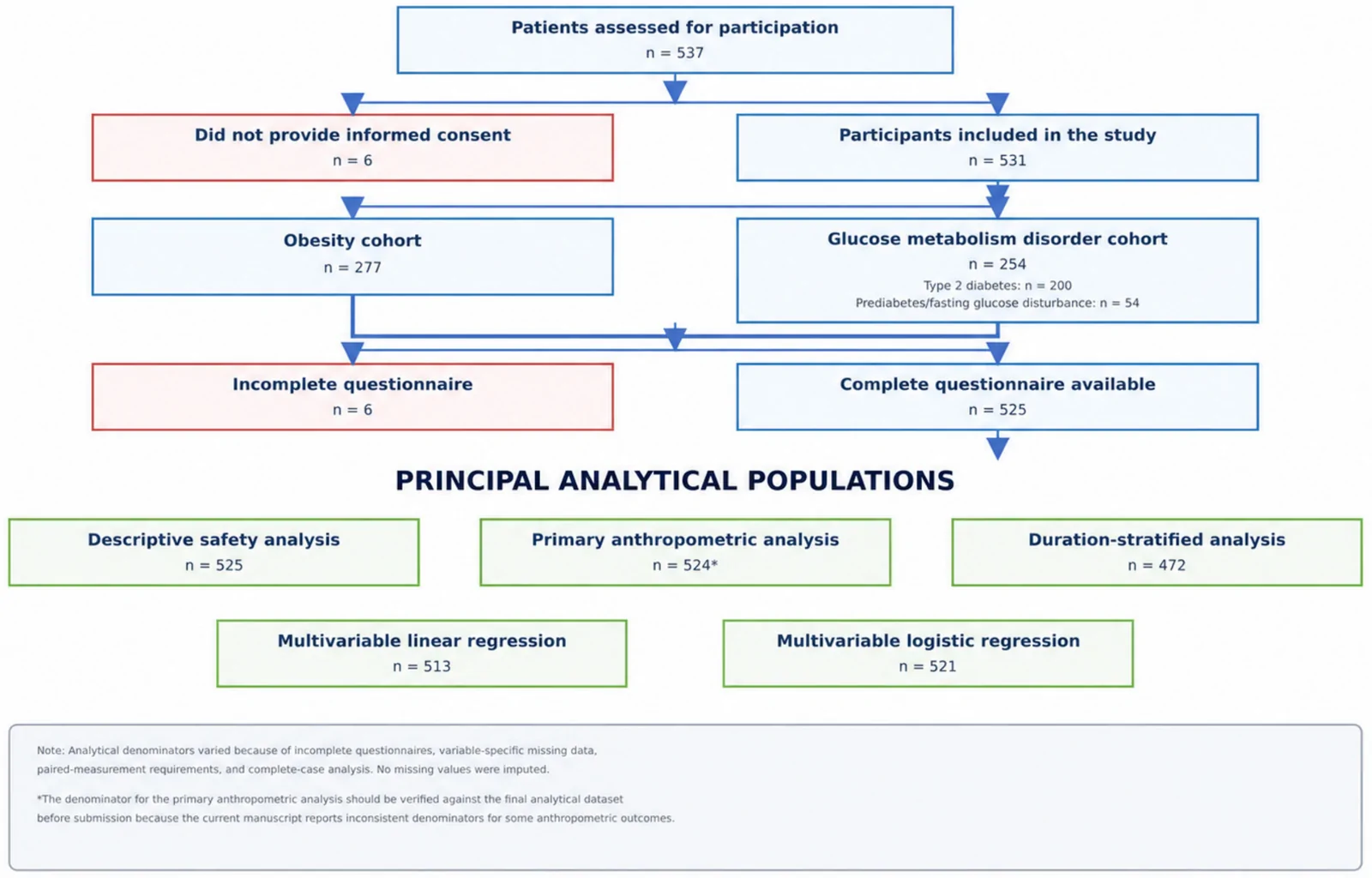
Flow diagram of participant recruitment, study inclusion, and analytical populations.

A total of 537 patients were initially recruited through participating community pharmacies. Six patients did not provide informed consent and were therefore excluded from the study. The remaining 531 participants constituted the final study population (Figure 1).

Participants were classified into two indication-based cohorts: the obesity cohort (*n* = 277, 52.2%; 95% CI: 47.9–56.4) and the glucose metabolism disorder cohort (*n* = 254, 47.8%; 95% CI: 43.6–52.1). Overall, 66.7% of participants were women (95% CI: 62.5–70.6), and the mean age was 56.5 ± 12.5 years. Mean height was 166.1 ± 9.0 cm, mean waist circumference was 113.21 ± 16.62 cm, and mean hip circumference was 113.7 ± 15.8 cm.

Hypertension was the most common comorbidity (45.8%; 95% CI: 41.6–50.0), followed by type 2 diabetes mellitus (37.7%) and dyslipidaemia (36.3%). Overall, 23.0% of participants reported no chronic comorbidities (95% CI: 19.6–26.7). Regarding lifestyle, 32.2% of participants reported not following a structured dietary plan, and 18.3% reported a sedentary lifestyle (Table 1).

### 3.2. Prescribing Patterns and Incretin Dosing Logistics

The most frequently prescribed medications were Mounjaro^®^ (34.1%; 95% CI: 30.2–38.3) and Ozempic^®^ (32.0%; 95% CI: 28.2–36.1), followed by Wegovy^®^ (17.3%) and Rybelsus^®^ (11.2%). Weekly administration was the predominant dosing schedule, reported in 86.1% of patients (95% CI: 82.9–88.8). At the time of the interview, treatment duration exceeded one year in 33.1% of patients and ranged between 6 and 12 months in 24.8%.

Overall, 72.2% of patients followed a gradual dose-escalation schedule (95% CI: 68.2–75.9), whereas 11.0% reported difficulties using the administration device. Prescriptions were issued more frequently by medical specialists (55.8%; 95% CI: 51.5–60.0), predominantly endocrinologists (80.3%; 95% CI: 74.8–84.2). Most treatments were prescribed through the public electronic health system (67.6%; 95% CI: 63.5–71.5) (Table 1).

As a sensitivity analysis, current dose was incorporated into the multivariable model together with treatment duration and the other prespecified covariates. Because dose units are molecule-specific, current dose was expressed relative to the maximum dose applicable to each treatment agent for this exploratory analysis. Current dose intensity was not independently associated with observed percentage weight loss (β = −1.38 percentage points, 95% CI −4.87 to 2.10; *p* = 0.436), whereas treatment duration remained strongly associated with observed weight loss.

However, current dose should not be interpreted as maximum achieved dose or cumulative exposure, as the study did not systematically capture the complete dose-escalation trajectory.

### 3.3. Observed Anthropometric Outcomes and Clinical Responsiveness Thresholds

A paired-sample *t*-test was performed to compare anthropometric measures between baseline and the study visit. Given that baseline weight was retrospectively recalled, these estimates represent observed weight changes and should not be interpreted as causal treatment effects. Mean body weight decreased by 12.17 ± 10.64 kg (95% CI: 11.25–13.09; *p* < 0.001), corresponding to a mean relative weight reduction of 11.71% ± 9.40% from baseline (95% CI: 10.90–12.53; *p* < 0.001). Mean BMI decreased from 36.13 ± 6.67 kg/m^2^ at baseline to 31.76 ± 6.02 kg/m^2^ at the study visit, representing a mean reduction of 4.40 ± 3.89 kg/m^2^ (95% CI: 4.06–4.73; *p* < 0.001).

Overall, 73.9% of patients (*n* = 383; 95% CI: 70.0–77.5) achieved a weight loss percentage of at least 5% from baseline, and 54.1% (*n* = 280; 95% CI: 49.7–58.3) achieved a weight loss percentage of at least 10% (Table 2).

Descriptively, the obesity cohort showed a greater observed percentage weight reduction than the glucose metabolism disorder cohort (14.24% ± 8.61% vs. 9.75% ± 9.91%, respectively). However, after adjustment for age, sex, treatment agent, dietary adherence, physical activity, treatment duration, and concomitant glucose-lowering therapies, the difference between cohorts was attenuated and was not statistically significant (adjusted β = 0.99 percentage points, 95% CI −1.24 to 3.22; *p* = 0.382).

The adjusted model included 513 participants and explained 30.8% of the variability in observed percentage weight loss (*n* = 513, R^2^ = 0.307, adj R^2^ = 0.281, Wald *F* = 19.51, *p* < 0.001).

Descriptively, the greatest mean absolute weight loss was observed with Wegovy^®^ (14.95 ± 9.52 kg), followed by Mounjaro^®^ (14.48 ± 10.24 kg), Saxenda^®^ (13.50 ± 16.26 kg), and Ozempic^®^ (10.60 ± 11.23 kg). However, no statistically significant differences were observed between treatments in percentage weight loss (ANOVA, *F* = 0.671; *p* = 0.672) or BMI reduction (*F* = 0.467; *p* = 0.832).

The assumption of homogeneity of variances was not violated for observed percentage weight loss (Levene’s test, W = 0.224, *p* = 0.925). Similar findings were observed for absolute weight loss (W = 0.841, *p* = 0.499) and BMI change (W = 0.878, *p* = 0.477). Accordingly, one-way ANOVA with Tukey’s HSD post-hoc comparisons was retained for the between-treatment analyses.

Treatment duration varied substantially across participants. Among participants with available treatment-duration data, 33 were at treatment initiation, 78 had been receiving treatment for ≤3 months, 110 for 3–6 months, 130 for 6–12 months, and 174 for >12 months.

Observed percentage weight loss differed across treatment-duration categories (one-way ANOVA, *p* < 0.001; Kruskal–Wallis test, *p* < 0.001). Mean observed percentage weight loss was 1.92% ± 4.13% among patients at treatment initiation, 7.54% ± 6.30% among those treated for ≤3 months, 11.19% ± 7.07% for 3–6 months, 14.96% ± 8.97% for 6–12 months, and 12.87% ± 10.88% for >12 months.

Diagnostic assessment indicated some departures from classical regression assumptions, including non-normality of residuals (Shapiro–Wilk W = 0.965, *p* < 0.001), heteroscedasticity (Breusch–Pagan LM = 36.88, *p* = 0.05), and potential model-specification concerns (Ramsey RESET F = 6.52, *p* = 0.011). The maximum VIF was 4.67, while the maximum Cook’s distance was 0.074. Full diagnostic results are provided in Supplementary Table S1.

Metformin was the most frequently used concomitant medication (25.0%), followed by SGLT2 inhibitors (11.5%) and insulin (9.6%). In unadjusted descriptive comparisons, observed percentage weight loss differed according to the use of some concomitant glucose-lowering therapies. However, these comparisons are potentially affected by confounding by indication and differences in the underlying clinical characteristics of patients with glucose metabolism disorder (metformin: 9.84% ± 9.63% vs. 12.32% ± 9.25%, *p* = 0.0107; SGLT2 inhibitors: 8.75% ± 9.07% vs. 12.07% ± 9.39%, *p* = 0.0101). Similarly, reductions in BMI were smaller among patients receiving metformin (*p* = 0.0168), SGLT2 inhibitors (*p* = 0.0043), or insulin (*p* = 0.0214) (Table 2). In the exploratory multivariable model, none of the concomitant glucose-lowering therapies was independently associated with observed percentage weight loss after adjustment for therapeutic indication, treatment agent, treatment duration, demographic characteristics, lifestyle variables, and the other concomitant therapies.

Because treatment duration varied considerably across participants, a duration-stratified sensitivity analysis was performed. Observed percentage weight loss increased across treatment-duration milestones, from 1.92 ± 4.13% among patients at treatment initiation to 7.54 ± 6.30% at ≤3 months, 11.19 ± 7.07% at 3–6 months, 14.96 ± 8.97% at 6–12 months, and 12.87 ± 10.88% among those treated for >12 months. The proportion achieving ≥5% weight loss was 10.0%, 58.2%, 83.7%, 87.8% and 75.3% respectively, while the corresponding proportions achieving ≥10% weight loss were 3.3%, 22.4%, 56.1%, 73.2%, and 60.5%. Overall differences in observed percentage weight loss across treatment-duration categories were statistically significant (one-way ANOVA, *p* < 0.001; Kruskal–Wallis *p* < 0.001).

A duration-stratified sensitivity analysis showed that observed weight loss varied across treatment-exposure categories. Mean observed relative weight loss increased from 1.92% at treatment initiation to 7.54% among patients treated for ≤3 months, 11.19% at 3–6 months, and 14.96% at 6–12 months, before reaching 12.87% among those treated for >12 months. The proportion achieving ≥5% weight loss ranged from 10.0% at treatment initiation to 87.8% among patients treated for 6–12 months, while the proportion achieving ≥10% ranged from 3.3% to 72.2%, respectively. Overall differences in observed percentage weight loss across treatment-duration categories were statistically significant (one-way ANOVA, F = 17.70, *p* < 0.001; Kruskal–Wallis H = 86.04, *p* < 0.001; Table 3).

These duration-stratified findings describe differences in cumulative observed weight change among patients with different treatment exposure durations and should not be interpreted as evidence of a causal treatment trajectory.

### 3.4. Pharmacovigilance and Exploratory Safety Analyses

Gastrointestinal symptoms were the most frequently reported suspected adverse drug reaction, affecting 63.0% of the study population. Nausea and vomiting were the most common events (32.6%), followed by constipation (19.8%). Asthenia was reported by 13.0% of patients, dizziness by 9.9%, musculoskeletal symptoms by 9.9%, and urinary disorders by 8.8%.

Safety analyses using crude odds ratios (ORs) identified several exploratory crude safety associations (Table 4).

**Figure 2:**
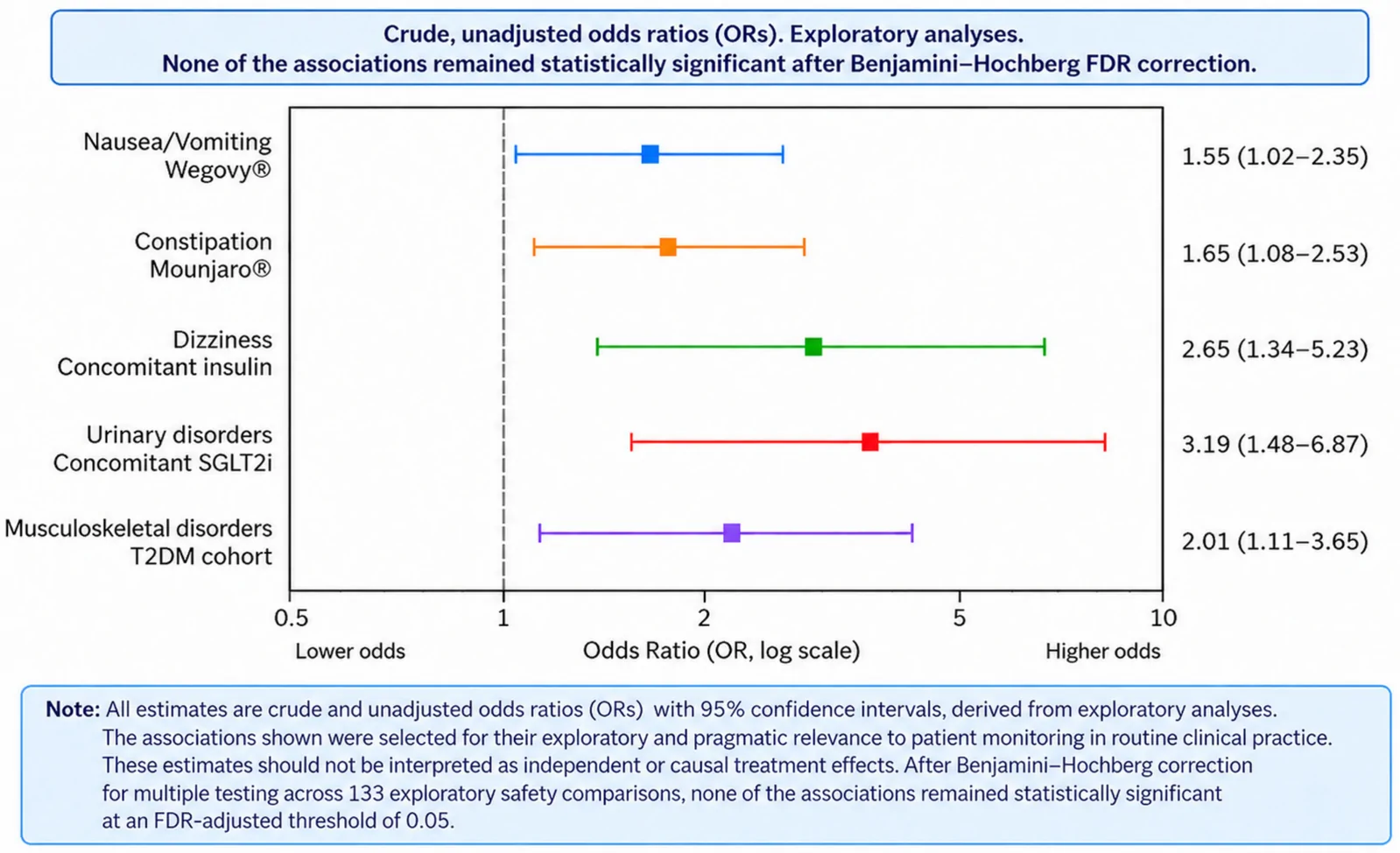
Exploratory crude associations between selected treatments, concomitant medications and reported adverse drug reactions.

The corresponding OR estimates and 95% confidence intervals are presented in Table 4 and Figure 2.

- Nausea and vomiting: A nominal crude association was observed between Wegovy^®^ and nausea/vomiting compared with the other treatments (OR = 1.55; 95% CI: 1.02–2.35). Trulicity^®^ (dulaglutide) was also associated with increased reporting of these events (OR = 2.01; 95% CI: 1.04–3.89). Higher reporting rates were observed among patients younger than 50 years (OR = 1.55; 95% CI: 1.07–2.23), whereas patients older than 70 years showed lower reporting rates (OR = 0.45; 95% CI: 0.24–0.83).
- Constipation: A nominal crude association was observed between Mounjaro^®^ and constipation (OR = 1.65; 95% CI: 1.08–2.53). Patients in the obesity cohort had higher reporting rates than those in the glucose metabolism disorder cohort (OR = 2.02; 95% CI: 1.29–3.17). Constipation was also more frequently reported between 3 and 6 months of treatment (OR = 1.60; 95% CI: 1.00–2.56).
- Dizziness: A nominal crude association was observed between concomitant insulin use and dizziness (OR = 2.65; 95% CI: 1.34–5.23). Patients younger than 40 years also showed higher reporting rates (OR = 2.54; 95% CI: 1.23–5.26).
- Musculoskeletal symptoms: Patients in the glucose metabolism disorder cohort were more likely to report musculoskeletal symptoms than those in the obesity cohort (OR = 2.01; 95% CI: 1.11–3.65). Treatment duration longer than one year (OR = 2.17; 95% CI: 1.22–3.86) and age over 70 years (OR = 2.01; 95% CI: 1.04–3.89) were also associated with higher reporting rates.
- Urinary disorders: Concomitant treatment with SGLT2 inhibitors was associated with increased reporting of urinary disorders (OR = 3.19; 95% CI: 1.48–6.87).
- Asthenia: No nominal exploratory crude associations were observed for asthenia, as all corresponding 95% confidence intervals included the null value.

The main exploratory crude safety association analyses are summarized graphically in Figure 2.

Among the 133 exploratory crude safety comparisons, 14 showed nominal statistical significance (*p* < 0.05). However, after applying the Benjamini–Hochberg procedure to control the false discovery rate, none of these associations remained statistically significant at an FDR-adjusted threshold of 0.05. Therefore, the crude associations were interpreted as exploratory patterns rather than confirmed drug-specific crude safety signals.

### 3.5. Exploratory Multivariable Analysis of Observed Percentage Weight Loss

The exploratory multivariable model, using HC3 heteroscedasticity-robust standard errors, showed statistically significant adjusted associations between observed percentage weight loss and treatment duration, treatment agent, dietary adherence, light physical activity, and age. The association with age was small and inverse. Detailed regression coefficients, 95% confidence intervals, and *p*-values are presented in Table 5. These findings should be interpreted cautiously given the cross-sectional design, retrospective ascertainment of treatment-initiation weight, and potential residual confounding by therapeutic indication and other measured or unmeasured factors. 

Increasing age was the only factor independently associated with a lower likelihood of reported suspected digestive adverse drug reactions. Each additional year of age was associated with approximately 3% lower odds of a digestive event (adjusted OR [aOR] = 0.97; 95% CI: 0.96–0.99; *p* = 0.003). No statistically significant independent associations were observed for sex, GLP-1RA agent, treatment duration, physical activity, dietary intervention, or concomitant glucose-lowering therapies after multivariable adjustment. A non-significant trend towards higher odds of digestive adverse drug reactions was observed among patients receiving treatment for 3–6 months (aOR = 2.21; 95% CI: 0.97–5.02; *p* = 0.059).

Model diagnostic assessment indicated departures from some classical regression assumptions, including non-normality of residuals and heteroscedasticity. The Ramsey RESET test also suggested potential model-specification concerns. Multicollinearity was not substantial, and no observations were excluded solely on the basis of diagnostic statistics. Full diagnostic results are provided in Supplementary Table S1.

### 3.6. Multivariable Associations with Reported Suspected Digestive Adverse Drug Reactions

The exploratory multivariable linear regression model included 513 participants (R^2^ = 0.307; adjusted R^2^ = 0.281; robust Wald F = 19.51, *p* < 0.001). Using HC3 heteroscedasticity-robust standard errors, treatment duration was significantly associated with observed percentage weight loss, with progressively greater observed weight loss among participants treated for longer periods compared with those at treatment initiation. Treatment group, dietary adherence, light physical activity, and age also showed statistically significant adjusted associations, whereas no nominal exploratory crude associations were observed for sex, moderate or intense physical activity, or concomitant SGLT2 inhibitor, metformin, insulin, or sulfonylurea use. Detailed estimates are presented in Table 6.

When independent associations were evaluated using multivariable logistic regression (Table 6), the crude association between Wegovy^®^ and suspected digestive adverse drug reactions was no longer statistically significant after adjustment for the covariates included in the model (adjusted OR = 1.69, 95% CI 0.88–3.25). Increasing age was the only variable that remained statistically significantly associated with reported suspected digestive adverse drug reactions, with each additional year associated with lower odds of a digestive event (adjusted OR = 0.97 per year, 95% CI 0.96–0.99; *p* = 0.003). However, the model showed limited overall explanatory ability (McFadden pseudo-R^2^ = 0.044).

These crude ORs should be interpreted as exploratory, hypothesis-generating patterns rather than clinically actionable risk estimates. Given their unadjusted nature and the absence of statistically significant associations after Benjamini–Hochberg correction, they should not be used for individual patient risk stratification or clinical decision-making.

Unlike adjusted regression models, which estimate the independent effect of each variable after controlling for confounding, crude ORs describe the overall probability of reporting an adverse drug reaction among patients exposed to a given treatment under routine clinical practice. The frequent contact between community pharmacists and patients receiving incretin-based therapies may provide an opportunity for structured monitoring and early identification of suspected adverse drug reactions. Whether pharmacist-led risk identification and targeted monitoring can improve clinical outcomes should be evaluated prospectively.

## 4. Discussion

This nationwide multicentre study provides a broad geographical perspective on the use of incretin-based therapies in Spanish community pharmacy practice. However, because participating pharmacies were recruited using convenience sampling, the study should not be considered nationally representative. The observed anthropometric outcomes and reported suspected adverse drug reactions provide complementary real-world information from routine community pharmacy practice, but the retrospective ascertainment of treatment-initiation weight and the cross-sectional design limit causal interpretation of observed weight changes.

### 4.1. Observed Weight-Loss Outcomes and Comparison with Clinical Trial Evidence

The weight-loss outcomes observed in this cross-sectional real-world sample are broadly consistent with findings reported in randomized clinical trials and other real-world studies of incretin-based therapies. In the present study, participants reported a mean reduction of 12.17 kg relative to body weight at treatment initiation. Because baseline weight was collected retrospectively, these findings should be interpreted as observed real-world weight-loss outcomes rather than causal estimates of treatment effectiveness. Moreover, because recalled baseline weight was not validated against an objective measurement, the resulting measurement error may have led to either overestimation or underestimation of the magnitude of weight loss. The direction and magnitude of this potential bias cannot be established from the present study. These results are clinically relevant and broadly consistent with those reported in the STEP, SURPASS, and SURMOUNT clinical programmes, although the magnitude of weight loss observed in routine clinical practice is generally lower than that reported in randomized trials [3,4,6,17,18,19].

Several factors may explain this difference. Unlike participants enrolled in clinical trials, patients treated in routine practice often receive lower maintenance doses, experience treatment interruptions, discontinue therapy because of adverse events or drug shortages, and show lower adherence to both medication and lifestyle recommendations. Together, these factors may contribute to the lower weight-loss outcomes commonly observed in routine clinical practice compared with randomized clinical trials [20,21].

Although the obesity cohort showed a greater observed percentage weight reduction than the GMD cohort in the descriptive analysis, this difference was substantially attenuated after multivariable adjustment and was no longer statistically significant. The adjusted mean difference was approximately 1 percentage point (β = 0.99; 95% CI −1.24 to 3.22; *p* = 0.382). This finding suggests that the descriptive difference between cohorts may be partly explained by differences in demographic characteristics, treatment exposure, treatment duration, lifestyle factors, and concomitant glucose-lowering therapies rather than by therapeutic indication alone. Given the observational design and the retrospective ascertainment of baseline body weight, this adjusted analysis should be interpreted as an association analysis rather than evidence of a causal difference in treatment response between obesity and GMD.

An important finding of the present study was the limited adherence to recommended lifestyle interventions. Approximately one-third of patients reported not following a structured dietary programme, and almost one in five reported a sedentary lifestyle. These findings reinforce current recommendations that pharmacological treatment should be accompanied by dietary modification and regular physical activity to maximise clinical benefit [22]. They also highlight the potential role of community pharmacists in reinforcing lifestyle advice, identifying barriers to adherence, and supporting patients throughout treatment.

This finding is consistent with previous studies suggesting that treatment response is influenced by multiple interacting clinical and behavioural factors that are difficult to capture using routinely collected baseline variables alone [23]. Rather than being determined by a single patient characteristic, weight-loss outcomes are likely to reflect the combined influence of treatment adherence, dose titration, lifestyle modification, treatment persistence, and individual biological variability.

Individual biological variability may also contribute to heterogeneity in treatment outcomes, particularly among patients with type 2 diabetes. Beyond treatment adherence, dose titration, treatment persistence, and lifestyle factors, genetic variation affecting insulin signalling and lipid metabolism may contribute to interindividual differences in metabolic susceptibility and treatment response. Candidate-gene studies have reported associations between variants in genes involved in lipid metabolism, including ABCA1 and LIPC, and type 2 diabetes susceptibility, while variants in INPPL1, which encodes SHIP2, a negative regulator of insulin signalling, have also been associated with type 2 diabetes in specific populations [24,25].

These findings provide a potential biological context for interindividual heterogeneity, but genetic variation was not assessed in the present study and such associations cannot be considered explanations for the observed weight-loss differences in our population.

Future studies integrating clinical, behavioural, treatment-exposure, and genetic or metabolic characteristics may help to better characterize the sources of interindividual variability in response to incretin-based therapies.

Treatment duration is an important contextual factor when interpreting the observed anthropometric outcomes. In the present study, patients at treatment initiation and those receiving therapy for more than 12 months were included within the same overall study population, reflecting the real-world spectrum of treatment exposure. As expected, observed percentage weight loss varied across treatment-duration categories, with progressively greater mean reductions during the first months of treatment and the highest observed mean reduction among patients treated for 6–12 months. Patients receiving treatment for >12 months showed a lower mean observed percentage reduction than the 6–12-month group, although this cross-sectional pattern cannot be interpreted as evidence of a plateau or subsequent attenuation of treatment effect. Differences in treatment duration, adherence, dose escalation, treatment discontinuation among non-responders, and other unmeasured factors may have contributed to the observed pattern.

Between-treatment comparisons should be interpreted cautiously because treatment groups differed not only in sample size but also in clinical indication and patient characteristics. As treatment allocation was not randomized, observed differences between treatment groups cannot be interpreted as causal treatment-specific effects.

### 4.2. Gastrointestinal Safety

Gastrointestinal adverse events were the most frequently reported adverse drug reactions in our study, affecting 63.0% of patients. This finding is consistent with evidence from randomized clinical trials and pharmacovigilance studies, in which gastrointestinal symptoms have consistently been identified as the most common adverse effects associated with GLP-1 receptor agonists [3,4,26]. In the STEP and SURMOUNT clinical programmes, gastrointestinal adverse events were reported in approximately 60–70% of participants, depending on the treatment regimen and dose-escalation schedule, supporting the consistency of our findings with previous evidence.

Although gastrointestinal adverse events were common across all treatments, differences in reporting patterns were observed between individual agents. Wegovy^®^ (semaglutide 2.4 mg) was associated with increased reporting of nausea and vomiting, whereas Mounjaro^®^ (tirzepatide) was more frequently associated with constipation. These differences may reflect pharmacodynamic differences between the two molecules, although the available evidence remains inconsistent and some systematic reviews have not identified clinically relevant differences in gastrointestinal tolerability [27,28].

The higher reporting of nausea and vomiting among patients receiving semaglutide may be related to the potent activation of central GLP-1 receptors involved in appetite regulation and the emetic response, particularly within the area postrema [29]. In our study, these events were reported more frequently among patients younger than 50 years, whereas lower reporting rates were observed in patients older than 70 years. Although the mechanisms underlying these age-related differences remain uncertain, previous studies have suggested that younger individuals may have greater sensitivity to GLP-1-mediated activation of central emetic pathways [27,29,30].

The interpretation of age as the only statistically significant independent predictor should nevertheless be considered in the context of the limited explanatory ability of the multivariable model (McFadden pseudo-R^2^ = 0.044). This indicates that the variables included in the model accounted for only a limited proportion of the variation in reported digestive adverse drug reactions. Consequently, the inverse association observed for age should be regarded as an exploratory, hypothesis-generating finding rather than as evidence that age is a major determinant of digestive tolerability. Other factors not captured or incompletely measured in the present study, including treatment titration, dose, comorbidity profile, previous gastrointestinal symptoms, adherence, and individual differences in symptom perception and reporting, may also contribute to the occurrence of reported digestive adverse drug reactions.

Conversely, constipation was more frequently reported among patients receiving tirzepatide and among those treated for obesity. Previous studies have suggested that the combined GIP/GLP-1 receptor agonist activity of tirzepatide may produce a more pronounced delay in gastrointestinal motility than selective GLP-1 receptor agonists, although evidence remains limited [26]. In addition, constipation was more frequently reported between three and six months after treatment initiation, suggesting that this period may represent an important window for monitoring gastrointestinal tolerability in routine clinical practice.

From a clinical perspective, these findings highlight the importance of proactive counselling by community pharmacists. Early identification of patients at increased risk of gastrointestinal adverse events, together with advice on hydration, dietary measures, gradual dose escalation, and symptom management, may improve treatment tolerability and support long-term treatment persistence.

To complement the exploratory crude safety association analysis, multivariable logistic regression models were developed to account for potential confounding factors. After adjustment, increasing age remained the only independent predictor associated with a lower probability of digestive adverse drug reactions, whereas the associations observed for individual GLP-1RA agents and concomitant therapies were attenuated. These findings suggest that part of the variability observed in the crude analyses may be explained by differences in patient characteristics rather than by the pharmacological agent itself. Although treatment duration of 3–6 months showed a trend towards an increased likelihood of digestive adverse events, this association did not reach statistical significance. Together, these results highlight the importance of complementing pharmacovigilance signal detection with multivariable analyses to distinguish true independent risk factors from associations potentially influenced by confounding.

- Although multivariable logistic regression provides the most appropriate approach for identifying independent predictors of adverse drug reactions by controlling for potential confounding factors, crude Odds Ratios remain highly relevant from a clinical and pharmacovigilance perspective. The two approaches address different but complementary research questions. Adjusted models estimate the independent association attributable to each exposure, whereas crude ORs quantify the overall probability of observing an adverse drug reaction among patients receiving a given treatment under routine clinical practice.
- Despite the absence of associations that remained statistically significant after FDR adjustment, crude odds ratios were retained because the purpose of these exploratory analyses was not to establish causal or independent drug-specific effects, but to characterize observed adverse reaction patterns in the real-world study population. These associations may therefore retain pragmatic value for prioritizing symptom assessment, patient counselling, and monitoring within the proposed community pharmacy dispensing protocol, while requiring cautious interpretation and external validation.

An unexpected finding was the inverse association between age and reported digestive adverse drug reactions, with older age independently associated with lower odds of digestive events. Several mechanisms may plausibly contribute to this observation. Age-related changes in gastrointestinal physiology and visceral sensory processing may alter the perception and reporting of gastrointestinal symptoms, potentially resulting in lower symptom recognition or reporting among older adults. In addition, treatment initiation and dose escalation may be more cautious in older patients in routine clinical practice, with slower titration or longer intervals between dose increases, potentially reducing the occurrence of dose-related gastrointestinal symptoms. Differences in treatment duration, concomitant medication use, and clinical monitoring may also contribute to this association, although these factors were accounted for only to the extent captured by the variables included in the multivariable model. Conversely, differential reporting cannot be excluded: older patients may be less likely to attribute mild gastrointestinal symptoms to incretin-based therapy or may underreport symptoms during pharmacy consultations. Therefore, the observed inverse association should be interpreted as a hypothesis-generating finding rather than evidence of a biological protective effect of age. Further prospective studies using standardized symptom assessment and formal adverse-event ascertainment are needed to determine whether this association reflects true differences in gastrointestinal tolerability or differences in symptom perception, treatment management, or reporting.

This distinction is particularly relevant for community pharmacy. Pharmacists implement patient-centred monitoring protocols based on the characteristics of the individual presenting at the point of care rather than on a hypothetical patient with confounding factors removed. Although crude ORs may provide descriptive, hypothesis-generating information regarding reporting patterns in routine practice, they should not be interpreted as estimates of individual patient risk, independent drug effects, or causal relationships. In this context, the crude ORs presented in Table 4 and Figure 2 should be interpreted as clinically useful measures for real-world risk stratification, whereas the adjusted analyses presented in Table 6 provide evidence regarding the independent contribution of each predictor.

### 4.3. Concomitant Medications and Safety

One of the most relevant findings of this study was the association between concomitant medications and the reporting of specific adverse events. Although GLP-1 receptor agonists have well-established gastrointestinal safety profiles, less attention has been paid to the influence of background therapies on tolerability in routine clinical practice.

Patients receiving concomitant insulin therapy were more likely to report dizziness than those not receiving insulin. Although the underlying mechanism cannot be established from the present study, this association may reflect episodes of mild hypoglycaemia resulting from the combined glucose-lowering effects of insulin and incretin-based therapies. This finding highlights the importance of reviewing insulin doses and reinforcing patient education, particularly during treatment initiation and dose escalation.

Similarly, concomitant treatment with SGLT2 inhibitors was associated with a higher reporting of urinary disorders. Given the well-established osmotic diuretic effect of SGLT2 inhibitors, these findings are biologically plausible and are consistent with the known pharmacological profile of this drug class [26,31]. Rather than representing an adverse effect attributable to GLP-1 receptor agonists alone, these symptoms may reflect the combined effects of both therapies in patients receiving combination treatment.

The observed differences in weight loss according to concomitant glucose-lowering therapy should be interpreted cautiously because of the potential for confounding by indication. Patients receiving metformin, SGLT2 inhibitors, or insulin are more likely to have type 2 diabetes and may differ systematically from patients not receiving these therapies in terms of disease duration, metabolic profile, treatment intensity, comorbidity burden, and baseline clinical characteristics. Therefore, the lower observed weight loss in some concomitant-treatment groups cannot be attributed to the concomitant medication itself. In particular, the comparison between patients receiving and not receiving insulin may reflect differences in diabetes severity and treatment complexity rather than an independent effect of insulin on weight loss. Similarly, differences observed among patients receiving metformin or SGLT2 inhibitors may partly reflect the underlying clinical characteristics associated with the indication for these therapies. These comparisons should therefore be regarded as descriptive and hypothesis-generating rather than causal.

Musculoskeletal symptoms were reported more frequently among patients with GMD and among those receiving treatment for more than one year. Several mechanisms may contribute to this finding. Patients with GMD frequently present with diabetic neuropathy, osteoarthritis, or other chronic musculoskeletal disorders that may increase symptom reporting independently of incretin therapy. In addition, rapid weight loss has been associated with reductions in lean body mass, particularly in the absence of adequate dietary protein intake and resistance exercise [21]. Although body composition was not assessed in the present study, this mechanism may partly explain the higher frequency of musculoskeletal symptoms observed in patients receiving long-term treatment.

The observation of musculoskeletal complaints among patients receiving longer-term treatment also warrants consideration in the context of potential lean mass loss. Although the present study did not directly assess skeletal muscle mass or body composition, substantial weight loss during incretin-based treatment may include reductions in lean mass in addition to fat mass. This may be particularly relevant in patients receiving treatment over extended periods, in whom preservation of muscle function and physical performance should be considered alongside weight reduction. From a clinical perspective, community pharmacists may have an important role in identifying patients potentially at risk of excessive lean mass loss during routine follow-up. This could include monitoring changes in body weight together with functional indicators such as strength, mobility, or physical performance, and referring patients for further clinical or nutritional assessment when clinically indicated. Pharmacists can also reinforce counselling on regular resistance exercise and adequate dietary protein intake, adapted to the patient’s age, clinical status, and overall nutritional needs. These measures may help support the preservation of muscle mass and function while maintaining the benefits of weight reduction.

Overall, these findings emphasise that the safety profile of incretin-based therapies should be interpreted within the context of the patient’s overall clinical condition and concomitant treatments. The frequent contact between community pharmacists and patients receiving incretin-based therapies may provide an opportunity for structured monitoring and early identification of suspected adverse drug reactions. Whether pharmacist-led risk identification and targeted monitoring can improve clinical outcomes should be evaluated prospectively.

The clinical relevance of GLP-1RA therapy in patients with obesity and GMD may extend beyond weight-related outcomes. These populations frequently present with overlapping metabolic comorbidities, including MASLD and chronic kidney disease, which may coexist and contribute to a broader cardiorenal-metabolic risk profile. Emerging evidence suggests that incretin-based therapies may influence kidney and liver outcomes in addition to their established metabolic effects. However, because renal function and hepatic status were not systematically characterized in the present study, these broader potential benefits could not be assessed. This reinforces the need for future community pharmacy-based real-world studies and monitoring frameworks to incorporate relevant renal and hepatic information when available.

The cardiovascular implications of T2DM may also extend beyond conventional metabolic and vascular risk factors to include alterations in autonomic and neurohumoral regulation. Experimental evidence suggests that T2DM may promote cardiac sympathetic overactivation through changes in signalling within the stellate ganglia. In a recent rat model of T2DM, activation of the PKC-α–MAPK14–ADAM17 pathway was accompanied by increased excitability of cardiac postganglionic sympathetic neurons and increased cardiac sympathetic nerve activity, providing a potential mechanistic link between the diabetic state and cardiovascular vulnerability [32]. Although these findings are experimental and cannot be directly extrapolated to the present population, they further illustrate the multifaceted cardiovascular pathophysiology associated with T2DM.

### 4.4. Study Strengths and Limitations

The main strength of this study is its nationwide scope and the inclusion of a large sample of patients recruited through community pharmacies across several autonomous communities in Spain. This multicentre design enhances the external validity of the findings by reflecting routine clinical practice and providing real-world evidence on the effectiveness and safety of incretin-based therapies. In addition, the study collected detailed information on prescribing patterns, lifestyle interventions, concomitant medications, and adverse drug reactions, allowing a comprehensive evaluation of treatment use in community pharmacy settings.

Although the study had nationwide geographical scope, participating pharmacies were recruited through convenience sampling and professional dissemination networks rather than probability-based sampling. The exact number of pharmacies receiving the study invitation and the number ultimately participating in the study could not be established because dissemination occurred through institutional and professional channels. Consequently, a pharmacy-level recruitment rate could not be calculated, and the participating pharmacies should not be considered a nationally representative sample of Spanish community pharmacies.

The study did not include a formal a priori statistical sample-size calculation; instead, an operational recruitment target of three patients per clinical indication cohort per participating pharmacy was established. Consequently, the study was not designed to achieve a prespecified statistical power for a specific effect size, and some subgroup and exploratory analyses may have limited precision and statistical power.

Although multivariable regression was used to adjust for measured covariates included in the model, residual confounding cannot be excluded. Relevant factors that were not measured or were incompletely captured in the study database may have influenced observed percentage weight loss. Therefore, the adjusted associations should not be interpreted as causal effects.

An important limitation is that baseline body weight was collected retrospectively based on patient recall and was not independently verified against clinical records or other objective sources. This introduces the possibility of recall bias and measurement error in the primary anthropometric outcome. Because the observed percentage weight loss was calculated using the retrospectively reported treatment-initiation weight as the denominator, inaccuracies in recalled baseline weight could affect both the magnitude and direction of the estimated relative weight change. The same limitation applies to the calculation of baseline BMI and the observed change in BMI. Misreporting of baseline weight could therefore lead to overestimation or underestimation of absolute weight loss, percentage weight loss, BMI change, and the proportion of participants classified as achieving clinically relevant thresholds of ≥5% or ≥10% weight loss.

This limitation may be particularly relevant because treatment duration varied substantially across participants, ranging from treatment initiation to more than 12 months. The time that elapsed between treatment initiation and study assessment may have influenced the accuracy of recalled baseline weight, although the magnitude and direction of any duration-related recall error cannot be established from the available data. In addition, the cross-sectional assessment does not allow the timing or trajectory of weight change to be independently verified. Consequently, the observed anthropometric differences should be interpreted as estimates based partly on retrospectively reported baseline values rather than as prospectively measured longitudinal changes. Future studies should prospectively record body weight at treatment initiation and at standardized follow-up intervals, ideally using calibrated measurement procedures, to provide more robust estimates of weight change over time.

An additional limitation is the absence of systematic renal and hepatic characterization. Kidney function parameters, including estimated glomerular filtration rate and albuminuria, as well as hepatic parameters relevant to metabolic dysfunction-associated steatotic liver disease (MASLD), were not systematically collected. This limits our ability to characterize the baseline cardiorenal and hepatic risk profile of the study population, to assess whether these conditions influenced treatment selection, or to explore their relationship with treatment outcomes and adverse drug reaction patterns. This is particularly relevant in patients with obesity and T2DM, in whom MASLD and chronic kidney disease frequently coexist and may share common metabolic and inflammatory pathways. Furthermore, emerging evidence suggests that incretin-based therapies may provide benefits extending beyond weight reduction and glycaemic control, including potential kidney and liver benefits in selected populations. Therefore, future real-world studies should incorporate systematic renal and hepatic characterization to better assess these broader clinical effects.

An additional limitation is the potential for selection and survivor bias arising from recruitment through community pharmacies during routine dispensing and pharmaceutical care activities. Although eligible patients were enrolled consecutively, inclusion required patients to remain engaged with treatment and to attend a participating pharmacy during the study period. Consequently, patients who discontinued treatment before the study assessment, particularly because of early intolerance, lack of effectiveness, or difficulties with treatment adherence, may have been less likely to be captured. This selective inclusion of patients who remained on treatment may have resulted in an overrepresentation of individuals with greater treatment persistence and potentially better tolerability, and could therefore have led to an overestimation of adherence and tolerability in the observed population. Conversely, early adverse reactions and treatment discontinuations may have been underestimated. Because patients who discontinued before recruitment were not systematically identified, the magnitude and direction of this potential survivor bias cannot be quantified from the present study. The findings should therefore be interpreted as reflecting patients who remained engaged with incretin-based therapy and community pharmacy follow-up during the study period, rather than all patients initiating treatment.

This potential survivor bias is particularly relevant when interpreting treatment persistence, adherence, and tolerability outcomes, as patients who discontinue early may differ systematically from those who remain in care. Future prospective studies including patients from treatment initiation and actively capturing treatment discontinuations are needed to provide less biased estimates of persistence, adherence, tolerability, and treatment outcomes.

A further limitation is that adverse drug reactions were identified through active pharmacist screening and patient self-report, without systematic application of a validated causality assessment tool such as the Naranjo algorithm or WHO-UMC criteria. Consequently, the study cannot establish definitive causal relationships between individual symptoms and incretin-based therapies. The safety findings should therefore be interpreted as patterns of reported suspected adverse drug reactions rather than confirmed drug-induced events.

Lifestyle variables, including adherence to treatment-related dietary recommendations and physical activity, were based on patient self-report. Although physical activity was assessed using a predefined threshold of ≥150 min per week and intensity categories accompanied by standardized examples, no validated physical activity instrument or objective activity measurement was used. Similarly, dietary adherence was not assessed using a validated dietary instrument. Consequently, reporting and classification bias cannot be excluded, and these variables may not fully capture the quantity, intensity, quality, or consistency of dietary and physical activity behaviours.

Confounding by indication is another important limitation of the analyses according to concomitant glucose-lowering therapies. Patients receiving metformin, SGLT2 inhibitors, insulin, or sulfonylureas may differ systematically from those not receiving these treatments in terms of diabetes status, disease severity, metabolic profile, comorbidity burden, and treatment complexity. Although these variables were considered where available in the study database, the observational cross-sectional design and the exploratory nature of the crude comparisons do not allow residual confounding to be excluded. Consequently, differences in observed weight loss according to concomitant medication use should not be interpreted as independent or causal effects of these therapies.

Finally, treatment duration varied considerably between participants, and residual confounding from unmeasured variables, including dietary habits, treatment adherence, socioeconomic factors, and other lifestyle characteristics, cannot be excluded. Furthermore, the absence of prospective follow-up limits the assessment of long-term effectiveness and safety outcomes.

Additionally, skeletal muscle mass, body composition, muscle strength, and physical performance were not systematically assessed; therefore, the present study cannot determine whether the reported musculoskeletal complaints were associated with lean mass loss or sarcopenia.

Despite these limitations, the study provides valuable real-world evidence on the use of incretin-based therapies in community pharmacy practice and identifies clinically relevant patterns that may help optimize patient monitoring and pharmaceutical care.

### 4.5. Implications for Community Pharmacy Practice and Future Research

The findings of this study have several implications for community pharmacy practice. In addition to characterizing real-world weight-loss outcomes among patients receiving incretin-based therapies, our results identified differences in adverse event reporting according to treatment, patient characteristics, and concomitant medications. These findings highlight the importance of individualized pharmaceutical care throughout treatment.

Community pharmacists are in a unique position to identify patients at increased risk of adverse events, reinforce adherence to dose-escalation schedules, promote lifestyle interventions, and provide counselling on the prevention and management of common gastrointestinal adverse effects. Routine follow-up during dispensing may also facilitate the early identification of treatment-related problems and support collaboration with prescribers when treatment adjustments are required.

The associations observed between specific therapies, concomitant medications, and adverse event reporting suggest that structured pharmaceutical follow-up could contribute to improving the safe use of incretin-based therapies in routine practice. These findings provide a rationale for the prospective development and evaluation of a standardized pharmacy-led dispensing and follow-up pathway. Such a protocol could incorporate patient education, assessment of concomitant medication, monitoring of treatment adherence and lifestyle measures, and early identification of adverse drug reactions.

Future prospective studies are needed to evaluate whether implementing a standardized pharmaceutical care protocol improves adherence, treatment persistence, adverse event management, and clinical outcomes in patients receiving incretin-based therapies.

**Figure 3:**
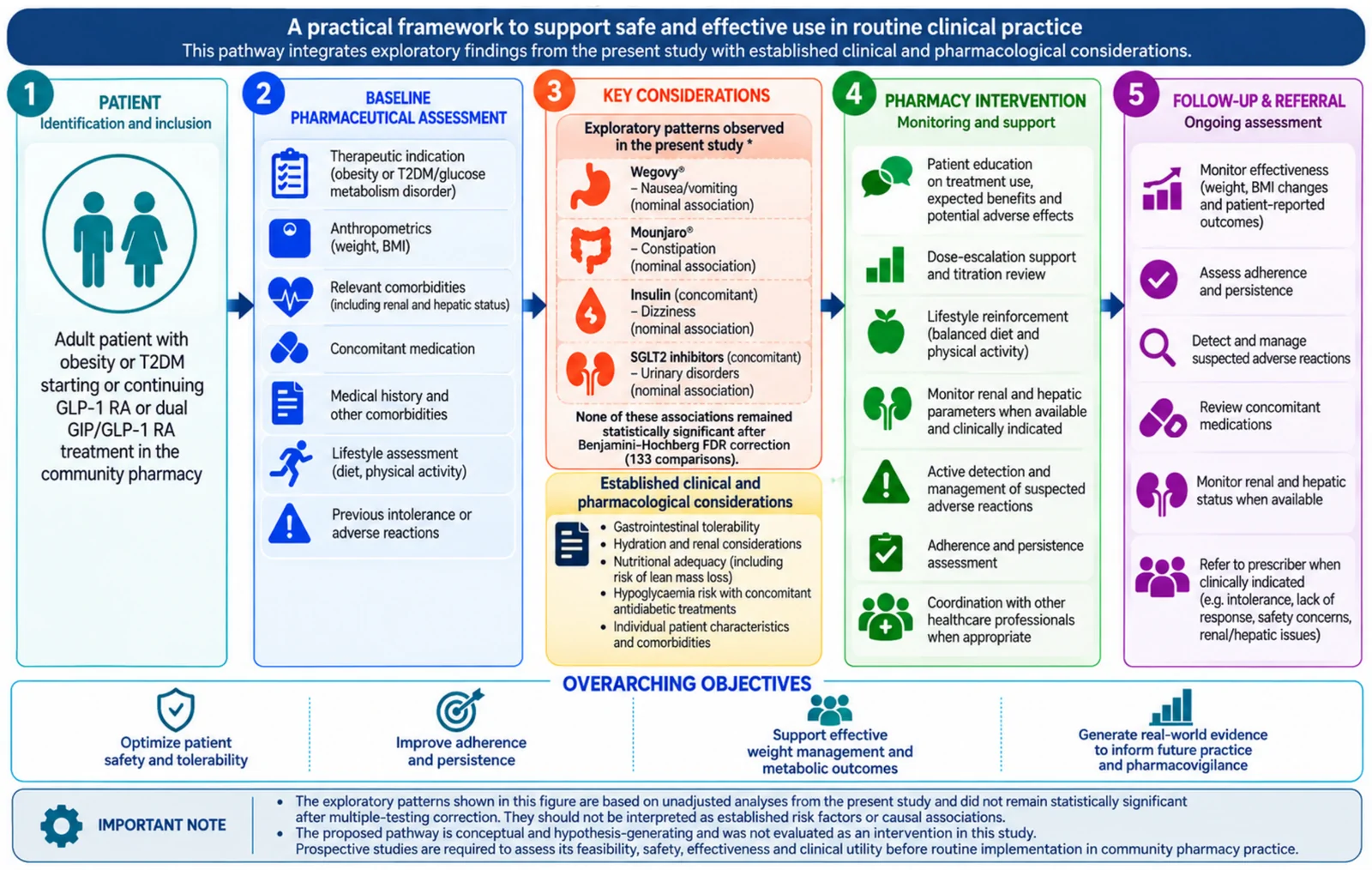
Conceptual community pharmacy pathway informed by exploratory patterns observed in the present study. The asterisk (*) identifies exploratory patterns derived from crude, unadjusted analyses conducted in the present study. The figure distinguishes exploratory patterns observed in the present study from established clinical/pharmacological considerations relevant to the monitoring of patients receiving incretin-based therapies. The exploratory patterns are derived from crude, unadjusted associations and should not be interpreted as established risk factors or drug-specific safety signals. Although several nominal associations were observed, none of the 133 comparisons remained statistically significant after Benjamini–Hochberg false discovery rate correction. The proposed pathway is conceptual and hypothesis-generating and was not evaluated as an intervention in the present study. Prospective studies are required to assess its feasibility, safety, effectiveness, clinical utility, and implementation before routine use in community pharmacy practice. Where available, the proposed monitoring framework should also consider documented chronic kidney disease, relevant renal function parameters, and known MASLD or other chronic liver disease. Community pharmacists are not expected to diagnose these conditions, but incorporating available clinical information may contribute to a more comprehensive assessment of treatment appropriateness, monitoring needs, and referral requirements.

Based on the findings of the present study, we propose a conceptual pharmacy-led dispensing and follow-up pathway for patients receiving incretin-based therapies (Figure 3). This framework integrates patient assessment, identification of potential safety risks, pharmaceutical counselling, and structured follow-up, providing a basis for future prospective evaluation.

## 5. Conclusions

In this nationwide multicentre community pharmacy study, substantial observed weight loss was recorded among patients receiving incretin-based therapies, based partly on retrospectively reported treatment-initiation weight. The proportion of participants achieving ≥5% and ≥10% observed weight loss was high, although these findings should be interpreted as real-world observed weight changes rather than causal estimates of treatment effectiveness. Differences in observed weight loss across treatment groups should be interpreted cautiously because of differences in treatment duration, therapeutic indication, sample size, and dose exposure.

Patient-reported suspected adverse drug reactions were frequently reported, particularly gastrointestinal symptoms. Although several nominal associations were identified in exploratory crude analyses, none remained statistically significant after Benjamini–Hochberg correction for multiple testing. These findings should therefore be interpreted as exploratory, hypothesis-generating patterns rather than clinically actionable risk estimates.

The frequent contact between community pharmacists and patients receiving incretin-based therapies may provide an opportunity for structured monitoring of treatment use, observed anthropometric outcomes, and patient-reported suspected adverse drug reactions. However, the present study did not evaluate a standardized pharmacy-led intervention or follow-up protocol. Prospective studies are therefore warranted to develop and evaluate whether standardized pharmacy-led dispensing and follow-up pathways can improve treatment monitoring, adherence, safety, or clinical outcomes.

## Figures and Tables

**Table 1:** Baseline demographic, clinical, comorbid, and prescribing characteristics of the study sample (*n* = 531).

Variable	Data Reporting Sub-Metrics & Statistical Boundaries
Continuous Variables (mean ± SD [Minimum–Maximum])	
Age (years) (*n* = 525)	56.48 ± 12.50 [19.0–85.0]
Stature (cm) (*n* = 524)	165.98 ± 9.24 [140.0–200.0]
Waist Circumference (cm) (*n* = 515)	113.21 ± 16.62 [70.0–180.0]
Hip Circumference (cm) (*n* = 513)	113.70 ± 15.83 [72.0–187.5]
Categorical Biological and Clinical Profile (*n* [%] [95% CI])	
Female Gender	350 (66.7%) [62.5–70.6]
Male Gender	175 (33.3%) [29.4–37.5]
Obesity Indication Cohort	277 (52.2%) [47.9–56.4]
Glucose Metabolism Disorder	254 (47.8%) [43.6–52.1]
Comorbidities (*n* [%] [95% CI])	
Hypertension	243 (45.8%) [41.6–50.0]
Diabetes Mellitus	200 (37.7%) [33.6–41.9]
Dyslipidemia	193 (36.3%) [32.4–40.5]
Cardiovascular Disease	84 (15.8%) [13.0–19.2]
Sleep Apnea	70 (13.2%) [10.6–16.3]
Prediabetes/Fasting Glucose Disturbance	54 (10.2%) [7.9–13.0]
None Reported	122 (23.0%) [19.6–26.7]
Concomitant Glucose-Lowering Medications (*n* [%] [95% CI])	
Metformin	133 (25.0%) [21.5–28.9]
SGLT2 inhibitors (Gliflozins)	61 (11.5%) [9.0–14.5]
Exogenous Insulin Formulations	51 (9.6%) [7.4–12.4]
Sulfonylureas	14 (2.6%) [1.6–4.4]
Lifestyle Interventions & Behavioral Adherence (*n* [%] [95% CI])	
Adherence to Structured Dietary Plans (Yes)	356 (67.8%) [63.7–71.7]
Non-adherence to Dietary Guidelines (No)	169 (32.2%) [28.3–36.3]
Weekly Physical Activity: Light (Walking, yoga, chores)	233 (44.4%) [40.2–48.7]
Weekly Physical Activity: Moderate (Brisk walking, cycling)	161 (30.7%) [26.9–34.7]
Weekly Physical Activity: Sedentary Lifestyle (No exercise)	96 (18.3%) [15.2–21.8]
Weekly Physical Activity: Intense (Competitive sports, aerobics)	35 (6.7%) [4.8–9.1]
Primary Incretin Dispensations and Titration Schemes (*n* [%] [95% CI])	
Mounjaro^®^ (Tirzepatide)	179 (34.1%) [30.2–38.3]
Ozempic^®^ (Semaglutide)	168 (32.0%) [28.2–36.1]
Wegovy^®^ (Semaglutide 2.4 mg)	91 (17.3%) [14.3–20.8]
Rybelsus^®^ (Oral Semaglutide)	59 (11.2%) [8.8–14.2]
Trulicity^®^ (Dulaglutide)	25 (4.8%) [3.2–6.9]
Saxenda^®^ (Liraglutide 3.0 mg)	2 (0.4%) [0.1–1.4]
Victoza^®^ (Liraglutide)	1 (0.2%) [0.0–1.1]
Adherence to Gradual Dose Escalation (Yes)	379 (72.2%) [68.2–75.9]
Exposure Chronology & Prescribing Framework (*n* [%] [95% CI])	
Long-term (>1 year)	174 (33.1%) [29.3–37.3]
6 to 12 months	130 (24.8%) [21.3–28.6]
3 to 6 months	110 (21.0%) [17.7–24.6]
0 to 3 months/Initiation Phase	111 (21.2%) [16.6–25.8]
Medical Specialist Prescription (Endocrinology leading)	293 (55.8%) [51.5–60.0]
National Health System Electronic Prescription	355 (67.6%) [63.5–71.5]

SD: Standard Deviation; CI: Confidence Interval. All qualitative stratification frequencies incorporate their corresponding data reporting denominators validated within the electronic registry logs. Note: The study population comprised 531 participants. Six participants did not complete the questionnaire and were excluded from questionnaire-based analyses. Additionally, isolated missing values for some variables resulted in slight variations in the denominator across analyses. Percentages were calculated using the number of participants with available data for each variable.

**Table 2:** Real-world anthropometric outcomes, weight-loss responder rates, and associations with treatment characteristics and concomitant medications.

Operational Evaluated Axis/Stratum	*N*	Absolute Weight Loss (kg), Mean ± SD	Absolute BMI Reduction (kg/m^2^), Mean ± SD	Relative Weight Loss (%), Mean ± SD	≥5% Weight Loss, *n* (%)	≥10% Weight Loss, *n* (%)
Overall study population	531	12.17 ± 10.64 ^a^	4.40 ± 3.89 ^a^	11.71 ± 9.40	383 (72.1%)	280 (52.7%)
Pharmacological brand						
Wegovy^®^	91	14.95 ± 9.52	5.41 ± 3.52	14.83 ± 8.42	78 (85.7%)	65 (71.4%)
Mounjaro^®^	179	14.48 ± 10.24	5.29 ± 3.81	13.93 ± 8.71	150 (83.8%)	112 (62.6%)
Ozempic^®^	168	10.60 ± 11.23	3.73 ± 3.99	9.80 ± 9.32	102 (60.7%)	75 (45.5%)
Trulicity^®^	25	7.87 ± 9.83	2.97 ± 3.65	7.69 ± 9.40	15 (60.0%)	9 (36.0%)
Rybelsus^®^	59	6.86 ± 8.86	2.46 ± 3.15	6.74 ± 9.47	35 (59.3%)	18 (30.5%)
Saxenda^®^	2	13.50 ± 16.26	5.85 ± 7.24	14.33 ± 16.94	1 (50.0%)	1 (50.0%)
Victoza^®^	1	3.80–	1.54–	5.21–	1 (100%)	0 (0%)
Clinical cohort						
Obesity cohort	276	13.79 ± 9.87	5.02 ± 3.67	13.43 ± 8.59	225 (81.5%)	171 (62.0%)
Glucose metabolism disorder cohort	243	10.33 ± 11.22	3.69 ± 4.03	9.74 ± 9.92	158 (65.0%)	110 (45.3%)
Exploratory comparisons by concomitant medication						
Metformin—exposed	133	10.53 ± 10.91	3.72 ± 3.83	9.84 ± 9.63	85 (63.9%)	54 (40.6%)
Metformin—non-exposed	398	12.71 ± 10.51	4.61 ± 3.88	12.32 ± 9.25	296 (74.4%)	225 (56.5%)
SGLT2 inhibitor—exposed	61	8.94 ± 10.31	3.17 ± 3.47	8.75 ± 9.07	35 (57.4%)	24 (39.3%)
SGLT2 inhibitor—non-exposed	470	12.57 ± 10.62	4.54 ± 3.91	12.07 ± 9.39	346 (73.6%)	255 (54.3%)
Insulin—exposed	51	8.94 ± 10.82	3.19 ± 3.80	9.24 ± 10.55	30 (58.8%)	20 (39.2%)
Insulin—non-exposed	480	12.51 ± 10.57	4.52 ± 3.88	11.95 ± 9.25	351 (73.1%)	259 (54.0%)
Sulfonylurea—exposed	14	13.52 ± 16.64	4.82 ± 5.84	12.27 ± 13.43	10 (71.4%)	8 (57.1%)
Sulfonylurea—non-exposed	517	12.12 ± 10.45	4.38 ± 3.83	11.67 ± 9.29	371 (71.8%)	271 (52.4%)

Mean ± SD: Mean ± Standard Deviation; CI: Confidence Interval; BMI: Body Mass Index; SGLT2i: Sodium-Glucose Cotransporter-2 Inhibitors. ^a^ Statistically significant difference between retrospectively reported baseline and study-assessment anthropometric measurements based on paired-sample *t*-tests (*p* < 0.001). ANOVA multi-comparison model utilizing Tukey HSD post-hoc testing yielded non-significant internal variations for brand-specific percentage weight loss (F = 0.671, *p* = 0.672) and absolute BMI reductions (F = 0.467, *p* = 0.832). Note: Saxenda^®^ (*n* = 2) and Victoza^®^ (*n* = 1) were excluded from drug-specific comparative analyses because of insufficient sample size. Note: The study population comprised 531 participants. Six participants did not complete the questionnaire and were excluded from questionnaire-based analyses. Additionally, isolated missing values for some variables resulted in slight variations in the denominator across analyses. Percentages and inferential analyses were calculated using all available observations for each variable. For paired analyses, participants were included only when both measurements required for the comparison were available. Note: Comparisons according to concomitant medication use were exploratory and unadjusted. Because concomitant medication use is related to underlying clinical indication and patient characteristics, these comparisons are susceptible to confounding by indication and should not be interpreted as independent or causal treatment effects. Note: Anthropometric outcomes were calculated using participants with available paired measurements. Therefore, cohort-specific denominators may be lower than the overall cohort sizes reported in Table 1.

**Table 3:** Duration-stratified sensitivity analysis of observed weight-loss outcomes.

Treatment Duration	*n*	Observed Weight Loss (kg), Mean ± SD	Observed Weight Loss (%), Mean ± SD	≥5% Weight Loss, n (%)	≥10% Weight Loss, n (%)	Paired *t*-Test, *p*-Value
Treatment initiation	30	2.18 ± 6.26	1.92 ± 4.13	3 (10.0)	1 (3.3)	0.066
≤3 months	67	7.55 ± 7.98	7.54 ± 6.30	39 (58.2)	15 (22.4)	<0.001
3–6 months	98	11.52 ± 7.84	11.19 ± 7.07	82 (83.7)	55 (56.1)	<0.001
6–12 months	115	15.26 ± 9.98	14.96 ± 8.97	101 (87.8)	83 (72.2)	<0.001
>12 months	162	13.97 ± 12.46	12.87 ± 10.88	122 (75.3)	98 (60.5)	<0.001

Overall comparison across treatment-duration categories: one-way ANOVA, F = 17.70, *p* < 0.001; Kruskal–Wallis test, H = 86.04, *p* < 0.001. Note: Weight loss was calculated as baseline body weight minus study-assessment body weight. Percentage weight loss was calculated relative to retrospectively reported treatment-initiation weight. Analyses were based on available paired anthropometric observations within each treatment-duration category. Treatment duration was recorded categorically as treatment initiation, ≤3 months, 3–6 months, 6–12 months, or >12 months. The paired-sample *t*-test assesses the observed difference between retrospectively reported baseline weight and study-assessment weight within each duration stratum; it should not be interpreted as evidence of a causal treatment effect.

**Table 4:** Odds Ratios for Selected Suspected Adverse Drug Reactions.

Risk Factor/Variable	Nausea & Vomiting	Constipation	Dizziness	Musculoskeletal	Urinary Disorders	Asthenia
	(OR [95% CI])	(OR [95% CI])	(OR [95% CI])	(OR [95% CI])	(OR [95% CI])	(OR [95% CI])
Pharmacological Brand						
Wegovy^®^	1.55 (1.02–2.35) *	1.35 (0.81–2.26)	0.51 (0.20–1.31)	0.62 (0.26–1.50)	0.85 (0.37–1.97)	0.92 (0.46–1.82)
Mounjaro^®^	0.82 (0.56–1.17)	1.65 (1.08–2.53) *	1.31 (0.73–2.34)	0.58 (0.30–1.13)	0.68 (0.34–1.35)	1.35 (0.81–2.26)
Ozempic^®^	1.03 (0.71–1.49)	0.67 (0.41–1.09)	1.12 (0.62–2.04)	1.44 (0.80–2.57)	1.13 (0.60–2.13)	0.76 (0.43–1.35)
Rybelsus^®^	0.81 (0.46–1.46)	0.40 (0.16–1.02)	0.48 (0.15–1.60)	1.65 (0.77–3.55)	1.42 (0.61–3.31)	1.20 (0.57–2.55)
Trulicity^®^	2.01 (1.04–3.89) *	0.59 (0.18–2.00)	2.12 (0.78–5.81)	2.12 (0.78–5.81)	1.39 (0.40–4.80)	0.61 (0.14–2.61)
Therapeutic Indication						
Obesity Cohort	1.12 (0.79–1.59)	2.02 (1.29–3.17) *	0.94 (0.53–1.66)	0.50 (0.27–0.90)	0.66 (0.36–1.22)	1.27 (0.76–2.11)
GMD Cohort	0.89 (0.63–1.26)	0.50 (0.32–0.77)	1.06 (0.60–1.88)	2.01 (1.11–3.65) *	1.51 (0.82–2.78)	0.79 (0.47–1.32)
Titration Protocol						
Gradual Escalation	1.35 (0.90–2.03)	1.52 (0.91–2.55)	1.62 (0.79–3.31)	1.84 (0.88–3.87)	0.98 (0.50–1.91)	2.00 (0.99–4.05)
Concomitant Therapies						
Insulin-treated	—	—	2.65 (1.34–5.23) *	—	—	—
SGLT2i (Gliflozins)	—	—	—	—	3.19 (1.48–6.87) *	—
Treatment Duration						
3–6 months	0.97 (0.63–1.49)	1.60 (1.00–2.56) *	1.53 (0.81–2.89)	0.68 (0.31–1.50)	1.19 (0.58–2.41)	1.46 (0.83–2.59)
Long-term (>1 year)	0.79 (0.54–1.15)	0.86 (0.54–1.35)	1.16 (0.64–2.10)	2.17 (1.22–3.86) *	1.30 (0.70–2.41)	0.78 (0.45–1.37)
Demographic Factors						
Under 40 years	1.47 (0.87–2.50)	1.35 (0.71–2.60)	2.54 (1.23–5.26) *	1.24 (0.50–3.05)	0.43 (0.10–1.83)	1.45 (0.68–3.09)
Under 50 years	1.55 (1.07–2.23) *	1.26 (0.80–1.99)	—	—	—	—
Over 70 years	0.45 (0.24–0.83) *	0.71 (0.37–1.36)	0.71 (0.30–1.73)	2.01 (1.04–3.89) *	0.98 (0.42–2.27)	0.73 (0.34–1.58)

* Nominally crude associations (unadjusted *p* < 0.05) are indicated by an asterisk. After Benjamini–Hochberg correction for multiple testing across 133 exploratory safety comparisons, none of the associations remained statistically significant at an FDR-adjusted threshold of 0.05. Data are expressed as crude ORs (95% CIs).

**Table 5:** Exploratory multivariable linear regression model for factors associated with observed percentage weight loss.

Variable	β	95% CI	*p*-Value
Age, per year	−0.065	−0.124 to −0.005	0.033
Female sex vs. male	1.202	−0.331 to 2.735	0.124
GLP-1RA agent			
Ozempic^®^ vs. Mounjaro^®^	−5.319	−7.458 to −3.180	<0.001
Wegovy^®^ vs. Mounjaro^®^	0.173	−1.672 to 2.019	0.854
Rybelsus^®^ vs. Mounjaro^®^	−7.468	−10.602 to −4.335	<0.001
Trulicity^®^ vs. Mounjaro^®^	−8.565	−12.807 to −4.322	<0.001
Dietary adherence			
Yes vs. no	2.569	0.823 to 4.315	0.004
Physical activity			
Light vs. sedentary	1.996	0.122 to 3.869	0.037
Moderate vs. sedentary	0.495	−1.384 to 2.375	0.605
Intense vs. sedentary	0.760	−2.009 to 3.528	0.591
Treatment duration			
≤3 months vs. initiation	5.203	2.734 to 7.673	<0.001
3–6 months vs. initiation	9.546	7.169 to 11.923	<0.001
6–12 months vs. initiation	13.760	11.401 to 16.119	<0.001
>12 months vs. initiation	14.452	11.821 to 17.082	<0.001
SGLT2 inhibitor	−1.407	−3.964 to 1.151	0.281
Metformin	0.912	−1.164 to 2.989	0.389
Insulin	−1.000	−4.160 to 2.160	0.535
Sulfonylurea	3.384	−3.409 to 10.178	0.329
Obesity cohort vs GMD cohort	0.994	−1.235 to 3.222	0.382

Model statistics: *n* = 513; R^2^ = 0.307; adjusted R^2^ = 0.281; robust Wald F = 19.51; *p* < 0.001. Note: β coefficients represent the estimated difference in observed percentage weight loss relative to the indicated reference category. HC3 heteroscedasticity-robust standard errors were used to calculate 95% confidence intervals and *p*-values. Mounjaro^®^ was used as the reference treatment. Treatment groups with fewer than five participants (Saxenda^®^ and Victoza^®^) were excluded from the multivariable analysis. The analysis was exploratory and does not establish causal relationships. Note: The obesity cohort was compared with the GMD cohort as the reference category. HC3 heteroscedasticity-robust standard errors were used. Saxenda^®^ and Victoza^®^ were excluded from multivariable analysis because of insufficient sample size.

**Table 6:** Multivariable logistic regression analysis of factors associated with suspected digestive adverse drug reactions.

Predictor	Adjusted OR (95% CI)	*p*-Value
Age, per year increase	0.97 (0.96–0.99)	0.003
Female sex vs. male	1.38 (0.93–2.05)	0.115
GLP-1RA agent		
Wegovy^®^ vs. Ozempic^®^	1.69 (0.88–3.25)	0.113
Mounjaro^®^ vs. Ozempic^®^	0.93 (0.54–1.61)	0.796
Rybelsus^®^ vs. Ozempic^®^	1.06 (0.57–1.98)	0.855
Trulicity^®^ vs. Ozempic^®^	0.74 (0.31–1.78)	0.499
Treatment duration		
≤3 months vs. initiation	1.56 (0.67–3.68)	0.305
3–6 months vs. initiation	2.21 (0.97–5.02)	0.059
6–12 months vs. initiation	1.64 (0.74–3.65)	0.226
>12 months vs. initiation	1.77 (0.78–4.01)	0.172
Physical activity		
Light vs. sedentary	0.90 (0.54–1.52)	0.701
Moderate vs. sedentary	0.76 (0.43–1.33)	0.336
Intense vs. sedentary	1.10 (0.44–2.76)	0.840
Dietary intervention, yes vs. no	1.05 (0.69–1.61)	0.810
Metformin use, yes vs. no	0.82 (0.50–1.34)	0.431
SGLT2 inhibitor use, yes vs. no	1.10 (0.59–2.03)	0.769
Insulin use, yes vs. no	1.08 (0.56–2.08)	0.808
Sulfonylurea use, yes vs. no	2.29 (0.66–7.93)	0.189

Model performance: *n* = 521; likelihood ratio χ^2^ = 25.4, *p* = 0.034; McFadden pseudo-*R*^2^ = 0.044. Note: OR, odds ratio; CI, confidence interval; GLP-1RA, glucagon-like peptide-1 receptor agonist; SGLT2, sodium–glucose cotransporter-2. Reference categories were male sex, Ozempic^®^ as the reference GLP-1RA, treatment initiation, sedentary physical activity, no dietary intervention, and absence of concomitant glucose-lowering medication. The model estimates adjusted associations with reported suspected digestive adverse drug reactions and does not establish causal relationships.

## Data Availability

The data supporting the findings of this study are not publicly available due to ethical and privacy considerations. However, anonymized data may be made available by the corresponding author upon reasonable request and subject to applicable ethical and data protection requirements.

## Supplementary Materials

The following supporting information can be downloaded at: https://www.mdpi.com/article/10.3390/healthcare14193355/s1, Table S1: Diagnostic assessment of the exploratory multivariable linear regression model for observed percentage weight loss.

## Abbreviations

The following abbreviations are used in this manuscript:

GLP-1	Glucagon-like peptide-1
GMD	Glucose Metabolism Disorder
GLP-1RAs	Glucagon-like peptide-1 Receptor Agonists
T2DM	Type 2 Diabetes Mellitus
GIP	Glucose-dependent Insulinotropic Polypeptide
BMI	Body Mass Index
ORs	Odds Ratios
MASLD	Metabolic Dysfunction-associated Steatotic Liver Disease
SGLT2i	Sodium-Glucose Cotransporter-2 Inhibitors
RWE	Real-world Evidence
eCRF	Electronic Case Report Form
MedDRA	Medical Dictionary for Regulatory Activities
ADR	Adverse Drug Reaction
SD	Standard Deviation
CI	Confidence Interval
ANOVA	Analysis of Variance
HSD	Honestly Significant Difference
aORs	Adjusted Odds Ratios
GDPR	General Data Protection Regulation
EU	European Union
cORs	Crude Odds Ratios
FDR	False Discovery Rate
