# Supplementary material for: Real-World Anthropometric Outcomes, Safety, and Use Patterns of GLP-1 and Dual GIP/GLP-1 Receptor Agonists in Obesity and Glucose Metabolism Disorder: A Nationwide Multicentre Community Pharmacy Study
Source: Healthcare (Basel). 2026 Oct 8;14(19):3355. doi: 10.3390/healthcare14193355 (PMC13665570; doi:10.3390/healthcare14193355)
Supplement: Supplementary file 1 [file healthcare-14-03355-s001.zip › healthcare-4554961-supplementary.pdf]

Supplementary Materials

Real-World Anthropometric Outcomes, Safety, and Use Patterns of GLP-1 and Dual GIP/GLP-1 Receptor Agonists in Obesity and Type 2 Diabetes: A Nationwide Multicentre Community Pharmacy Study

Olatz Vergniory-Trueba and Carlos Treceño-Lobato

Table S1. Diagnostic assessment of the exploratory multivariable linear regression model for observed per-centage weight loss.

**Diagnostic assessment**	**Result**
Number of observations	513
Shapiro-Wilk test for residual normality	W = 0.965; p < 0.001
Breusch-Pagan test for homoscedasticity	LM = 36.88; p = 0.005
Ramsey RESET test for model specification	F = 6.52; p = 0.011
Maximum VIF	4.67
Maximum Cook's distance	0.074
Maximum leverage	0.128
Maximum externally studentized residual	6.02

Note: No observations were excluded solely on the basis of regression diagnostic statistics. HC3 heteroscedasticity-robust standard errors were used for inference because heteroscedasticity was detected.

Abbreviations: HC3, heteroscedasticity-consistent covariance matrix estimator type 3; RESET, Regression Equation Specification Error Test; VIF, variance inflation factor.
